# Bempedoic Acid for Lipid Management in the Indian Population: An Expert Opinion

**DOI:** 10.7759/cureus.35395

**Published:** 2023-02-24

**Authors:** Jagdish Hiremath, J C Mohan, Prakash Hazra, JP S Sawhney, Ashwani Mehta, Sadanand Shetty, Abraham Oomman, Mahesh K Shah, Ganapathi Bantwal, Rajeev Agarwal, Rajiv Karnik, Peeyush Jain, Saumitra Ray, Sambit Das, Vibhuti Jadhao, Sachin Suryawanshi, Hanmant Barkate

**Affiliations:** 1 Cardiology, Ruby Hall Clinic, Pune, IND; 2 Cardiology, Jaipur Golden Hospital, Jaipur, IND; 3 Cardiology, Apollo Clinic Hospitals, Ballygunge, Kolkata, IND; 4 Cardiology, Sir Ganga Ram Hospital, New Delhi, IND; 5 Cardiology, Sadanand Healthy Living Center (P) Ltd. Sion (East), Mumbai, IND; 6 Cardiology, Apollo Hospitals, Chennai, IND; 7 Cardiology, MK’s Heart Care, Vile Parle, Mumbai, IND; 8 Endocrinology, Diabetes and Metabolism, St. John's Medical College and Hospital, Bengaluru, IND; 9 Cardiology, Jaswant Rai Specialty Hospital, Meerut, IND; 10 Cardiology, Dr. Karnik’s Cardiac Clinic, Mulund West, Mumbai, IND; 11 Cardiology, Fortis-Escorts Heart Institute and Research Centre, Delhi, IND; 12 Cardiology, Woodlands Multispeciality Hospital, Kolkata, IND; 13 Endocrinology, HiTech Medical College and Hospitals, Bhubaneshwar, IND; 14 Global Medical Affairs, Glenmark Pharmaceuticals Limited, Mumbai, IND

**Keywords:** india, c-reactive protein, cardiovascular disease, lipid lowering agents, bempedoic acid

## Abstract

Lipid-lowering is a central theme in the management of patients with atherosclerotic cardiovascular disease (ASCVD) and heterozygous familial hypercholesterolemia (HeFH), with statins being currently used as the first-line lipid-lowering agent (LLAs). Bempedoic acid (BA) has been recently approved for lipid management in ASCVD/HeFH patients. This expert opinion paper brings out the essential concept to assess the current place of BA in the Indian population. Here we highlight that the majority of the patients with clinical ASCVD may not be receiving the optimal dose of statin, thereby failing to achieve their lipid targets. The addition of BA to statin results in a significant reduction in low-density lipoprotein cholesterol (LDL-C) along with substantial reductions in non-high-density lipoprotein cholesterol (non-HDL-C), apolipoprotein B (ApoB), and high-sensitivity C-reactive protein (hsCRP) levels. For patients who do not achieve LDL-C targets, BA can be an effective add-on alternative to choose among non-statin LLAs. BA is a good choice for statin-intolerant cases, especially in combination with ezetimibe. Given the lack of effect of worsening hyperglycemia or any increase in the occurrence of new-onset diabetes, BA can be used without hesitation in patients with diabetes. The small risk of hyperuricemia could be mitigated with appropriate patient selection and monitoring of serum uric acid levels in patients at high risk of hyperuricemia. We believe BA is an excellent non-statin therapy that is efficacious, well-tolerated, and cost-effective for lipid management in ASCVD, HeFH, and statin-intolerant patients in India.

## Introduction and background

Hydroxy-methyl-glutaryl-CoA inhibitors (HMG-CoA inhibitors) or statins are the principal lipid-lowering agents (LLAs) currently in use for the primary and secondary prevention of atherosclerotic cardiovascular diseases (ASCVD) [1,2]. Despite the use of statin in optimal doses, target low-density lipoprotein cholesterol (LDL-C) levels may not be achieved in all patients [3-5]. Thus, the use of non-statin LLAs such as ezetimibe, proprotein convertase subtilisin/kexin type 9 (PCSK9) inhibitors, and bile acid sequestrants becomes necessary to further lower the LDL-C, especially among patients with a high risk of ASCVD [6]. Besides the non-attainment of LDL-C targets, statin intolerance (SI) also often necessitates the initiation of non-statin LLAs [7]. Recently, bempedoic acid (BA), a new non-statin LLA, that inhibits the enzyme adenosine triphosphate (ATP)-citrate lyase (ACL), has been approved as an adjuvant to maximally tolerated statin therapy to lower LDL-C in patients with ASCVD and heterozygous familial hypercholesterolemia (HeFH) [8,9]. Being orally administered, BA is an attractive alternative to existing non-statin LLAs and has been found to be safe and effective in various clinical trials in terms of lipid-lowering and reduction in high-sensitivity C-reactive protein (hsCRP) [10-13]. The current cholesterol management remains LDL-centric and therefore BA has a potential role in the management of ASCVD. Given the availability of ezetimibe as possible add-on therapy to statins, the current role of BA needs to be well-defined. To simplify the dilemma of choice among non-statin LLAs after statin and in statin-intolerant patients, this expert opinion paper was developed to examine and define the precise role of BA in lipid management.

## Review

2. Bempedoic acid: pharmacology and clinical evidence

2.1. Indication

BA is approved in the United State and Europe for use in adults with primary hypercholesterolemia (heterozygous familial and non-familial) or mixed dyslipidemias, or established ASCVD patients who require additional lowering of LDL-C as an adjunct to diet in combination with a statin or statin with other LLAs with the maximum tolerated dose of a statin. It is also indicated either alone or in combination with other LLAs in patients who are statin-intolerant, or for whom statins are contraindicated [14,15].

2.2. Pharmacology

BA is a novel non-statin LLA that inhibits the enzyme ACL. BA is a prodrug that is converted to bempedoyl CoA, its active form, by the enzyme very long-chain acyl-CoA synthetase-1 (ACSVL1). As the liver and kidneys mainly express this enzyme, the activity of BA is restricted to the liver. ACL is responsible for the cleaving of citrate from mitochondria into oxaloacetic acid and acetyl-CoA. Inhibition of ACL by BA in the liver results in reduced levels of acetyl-CoA. This in turn causes LDL-C receptor upregulation on hepatic cells resulting in increased reuptake of LDL particles in the liver, thereby reducing the plasma LDL-C levels. The activity of active metabolite is limited to the liver and therefore may not result in an effect on skeletal muscles observed with statins. The unique mechanism of BA is complementary to the action of other LLAs, making it a suitable agent for use in combination with other available LLAs [9,14].

BA at a dose of 180 mg per day achieved maximum concentration in plasma (C_max_) after 3.5 hours and a steady state is achieved after seven days. Food has no effect on its bioavailability. It is bound to plasma proteins (99.3% parent drug, 98.8% glucuronide metabolite, and 99.2% active metabolite). The liver is the primary site of metabolism. Excretion happens mainly via kidneys (70% of parent drug and metabolites) whereas 30% is via the fecal route. No dosage adjustments are necessary for mild or moderate renal or hepatic (Child-Pugh class A or B) impairment. There is limited data on BA in patients with severe renal impairment (eGFR <30 mL/min/1.73 m^2^) and it has not been studied in patients with end-stage renal disease (ESRD) receiving dialysis. Age, sex, race, or weight does not affect the pharmacokinetic parameters of BA. When using BA, concomitant use of simvastatin in doses above 20 mg is contraindicated. Concomitant administration of BA and simvastatin may increase the area under the curve (AUC) and C_max_ of simvastatin. Inhibition of renal tubular organic anion transporter 2 (OAT2) is responsible for the increase in serum uric acid [9,14,15].

2.3. Clinical Evidence

BA has been evaluated in various randomized clinical trials. The CLEAR (Cholesterol Lowering via Bempedoic acid, an ACL-Inhibiting Regimen) trials involved phase 3 trials to study the efficacy and safety of BA in patients of ASCVD or HeFH who were either on maximally tolerated statins or lower-dose/no-statins therapy. We briefly discuss the evidence from these trials. Table 1 shows the mean percentage reductions in various lipid parameters in the BA group. 

**Table 1:** Mean percent reduction in lipid parameters with bempedoic acid use at 12 weeks in CLEAR trials All the reductions are statistically significant with p<0.05 ASCVD: atherosclerotic cardiovascular disease; HeFH: heterozygous familial hypercholesterolemia; LDL-C: low-density lipoprotein cholesterol; HTN: hypertension; DM: diabetes mellitus; hsCRP: high-sensitivity C-reactive protein; HDL-C: high-density lipoprotein cholesterol; ApoB: apolipoprotein B

Studies	Population	Number and distribution of patients	Study duration	Intervention	Mean % reduction in bempedoic acid group at 12 weeks in lipid parameters				
					LDL-C	hsCRP	Non-HDL-C	Total cholesterol	ApoB
CLEAR Harmony [10]	ASCVD and/or HeFH; taking maximally tolerated statin with or without other lipid-lowering agents; LDL-C ≥70 mg/dL	N=2230, HTN 79%, DM 29%, HeFH 4%, ASCVD 98%	52 weeks	Bempedoic acid 180 mg (n=1488) versus placebo (n=742)	-16.5%	-22.4%	-11.9%	-10.3%	-8.6%
CLEAR Wisdom [11]	ASCVD and/or HeFH; taking maximally tolerated statin; LDL-C ≥ 100 mg/dL	N=779, ASCVD 95%, HTN 85%, DM 30%, HeFH 5%	52 weeks	Bempedoic acid 180 mg (n=522) versus placebo (n=257)	-15.1%	-18.7%	-10.8%	-9.9%	-9.3%
CLEAR Serenity [12]	History of statin intolerance to ≥2 statins; LDL-C ≥130 mg/dL for primary prevention, LDL-C≥ 100 mg/dL for secondary prevention in ASCVD or HeFH	N=345, DM 26%, HTN 68%, ASCVD 39%, HeFH 2%	24 weeks	Bempedoic acid 180 mg (n=234) versus placebo (n=111); using other lipid-lowering agents: 30%; using low-dose statin: 10%; never used lipid-lowering agent: 57%	-23.6%	-25.4%	-19.0%	-16.1%	-15.5%
CLEAR Tranquility [13]	History of statin intolerance with or without using low-dose statin; LDL-C ≥100 mg/dL	N=269, DM 19%, HTN 60%	12 weeks	Bempedoic acid 180 mg plus ezetimibe 10mg (n=181) versus placebo plus ezetimibe (n=88); using low-dose statin: 30%	-23.5%	-32.5%	-18.4%	-15.1%	-14.6%

CLEAR Harmony [10] was another phase 3 study with a similar population to that of CLEAR Wisdom. At week 12 of therapy, the incidence of total adverse events (AEs) (78.5% vs. 78.7%) and serious AEs (14.5% vs. 14.0%) was comparable in the two groups. The incidence of AEs leading to treatment discontinuation was higher with BA than with placebo (10.9% vs. 7.1%). Gout was more frequent with BA than with placebo (1.2% vs. 0.3%). The mean reduction in the LDL-C was significantly higher with BA than with placebo at 12 weeks (difference between the groups: -18.1%, p<0.001) and 24 weeks (difference between the groups: -16.1%, p<0.001). At the end of 52 weeks also, the LDL-C reduction effect was persistent (difference between the groups: -12.6%, p<0.001). Similarly, the change in non-high-density lipoprotein cholesterol (non-HDL-C), total cholesterol (TC), apolipoprotein B (ApoB), and hsCRP was significant at all time points with BA when compared to the placebo. The open-label extension of CLEAR Harmony [16] to 78 weeks assessed the efficacy and safety of BA. In the BA group of the parent study, the effects observed of LDL-C and other parameters remained stable through 130 weeks of treatment. With the initiation of BA in 492 patients in the placebo group of the parent study, there was a reduction in LDL-C and other parameters, consistent with previous observations. In the open-label extension period, the treatment-emergent AEs and AEs of special interest remained comparable in patients whether treated for 130 weeks or 78 weeks (78% vs. 78%). These results demonstrate consistent efficacy and better tolerability of BA over a two-and-half-year period.

In the CLEAR Wisdom trial [11], 779 patients with ASCVD, HeFH, or both who had LDL-C levels >70 mg/dl despite maximally tolerated LLAs were randomized to BA (180 mg) (n=522) or placebo (n=257) once daily. At 12 weeks, the reduction in LDL-C, non-HDL-C, TC, and ApoB was significant with BA when compared to the placebo. Reduction in hsCRP (mean percent reduction: -18.7%) was also significant. 

In the CLEAR serenity [12] trial, 354 patients with hypercholesterolemia with a history of intolerance to at least two statins (one at the lowest available dose) were studied. At baseline, 93% of patients had statin-associated muscle symptoms (SAMS). At week 12, the reduction in LDL-C was significant with BA when compared to the placebo (placebo-corrected difference: -21.4%; p<0.001). Drug-related AEs (21.8% vs. 18.0%) were comparable. Muscle-related AEs were also comparable with BA than with the placebo group (12.8% vs. 16.2%). Another similar trial, the CLEAR Tranquility [13], recruited patients who had SI. Patients had a four-week run-in period of ezetimibe 10 mg/d (E10) after which they were randomized to receive 180 mg of BA (n=181) or placebo (p=88) in addition to E10. At 12 weeks, the LDL-C reduction was significant with BA (difference between the groups: -28.5%, p<0.0001). Similarly, significant reductions in non-HDL-C (-23.6%), TC (-18.0%), ApoB (-19.3%), and hsCRP (-31.0%) were observed with BA vs. placebo (p<0.001). The incidence of AEs was similar (48.6% vs. 44.8%) as was the incidence of AEs leading to discontinuation (6.1% vs. 5.7%). Hyperuricemia was higher with BA than with placebo (7.7% vs. 2.3%) whereas myalgia rates (1.7% vs. 2.3%) were similar.

A pooled analysis [17] of four trials was conducted involving 3623 patients randomized to BA (n=2425) or placebo (n=1198). At 12 weeks, the mean LDL-C change in patients with ASCVD, HeFH, or both and those with SI was significant with BA (difference between the groups: -17.8% and -24.5%, respectively; p<0.001 for both patient group comparisons). The effect persisted till week 52 (-12.7%) in patients with ASCVD, HeFH, or both and till week 24 weeks (-22.2%) in patients with SI. The increase in uric acid levels (2.1% vs. 0.5%) and gout (1.4% vs. 0.4%) were more frequent with BA. Compared to the placebo, the 12-week reduction in non-HDL-C cholesterol was significant with BA in ASCVD/HeFH patients (-13.1%) and in statin-intolerant (-20.4%) patients. Similarly, reduction in ApoB (-12.1% and -16.9%) and reduction in hsCRP (-18.1% and -27.4%) were significant with BA in ASCVD/HeFH and statin-intolerant patients, respectively. Thus, the current evidence with BA indicates that when added to maximally tolerated lipid-lowering therapy (LLT) (high- or moderate-statin or no statin), BA significantly reduces LDL-C with an acceptable safety profile. In addition, a significant reduction in non-HDL-C cholesterol, ApoB, and hsCRP is also observed with BA. 

Another post-hoc analysis of pooled data from phase 3 clinical trials [18] analyzed changes in glycemia levels categorized by the baseline glycemic status. The annual rate of new-onset diabetes in patients with normoglycemia (n=618, 0.3% vs. 0.8%) and prediabetes (n=1868, 4.7% vs. 5.9%) at baseline was lower in BA than in placebo. Compared to placebo, there was a significant reduction in the HbA1c with BA among patients with diabetes (-0.12%, p<0.0001) and prediabetes (-0.06%, p<0.0001) at baseline. The LDL-C reduction was consistent across the baseline glycemic groups. A meta-analysis substantiated this finding further with the demonstration of a significant reduction in new-onset or worsening diabetes risk (odds ratio: 0.66, 95% confidence interval: 0.48-0.90) [19].

The CLEAR outcomes trial is underway to enroll 14,014 patients who have ASCVD or are at high risk of ASCVD with documented SI, and with LDL-C levels ≥100 mg/dL despite maximally-tolerated LLT. This trial will establish whether BA reduces the incidence of adverse CV events in the studied population when compared to the placebo [20]. On December 7, 2022, Esperion Therapeutics Inc. released a press note stating that BA (NEXLETOL®) demonstrated a statistically significant reduction in four-point major adverse CV event (MACE) [21]. This is welcoming and opens up a new strategy for reducing the ASCVD outcomes among patients with ASCVD and HeFH.

3. Need and objective of the expert opinion

Indian population is considered one of the “high-risk” ethnic groups for the development of ASCVD. Dyslipidemia in Indians is different from the Western populations in terms of a higher prevalence of high LDL-C, low HDL-C, and high triglycerides (TGs) [22]. In addition, Indians have a higher likelihood of increased lipoprotein (a) [Lp(a)] and high levels of ApoB contributing to the ASCVD burden [23]. Currently, ezetimibe is the principal add-on therapy to statin for patients who do not achieve target LDL-C levels [24]. The use of PCSK9 inhibitors is largely reserved for individuals who fail to achieve LDL-C targets despite combination therapy and optimal lifestyle therapy [24]. Findings from a cross-sectional survey of 400 physicians across India with specialization in internal medicine (55%), cardiology (5.5%), diabetes, endocrinology or nephrology combined (7%) or MBBS (26.1%) indicated the use of statins and ezetimibe as major LLT [24]. Besides, due to the limited options for dyslipidemia, its management is more challenging, especially in patients with SI [25]. A meta-analysis identified that the SI prevalence of 4.9% to 17% is reported in various RCTs and cohort studies [26]. This is in accordance with the opinion of Indian physicians who reported an SI prevalence of <20%. In practice, reducing statin dose or switching to another statin remains the major approach with the use of a non-statin drug in those who do not tolerate low-dose statin/alternate statin [24]. Thus, current lipid management relies on statin and ezetimibe with limited options for treating those with SI. BA has emerged as an important statin add-on alternative. However, its current place in management remains unclear. This expert opinion has been developed with the objective to guide physicians to effectively use BA in the armamentarium of current lipid-management strategies.

3.1. Obtaining Expert Opinion

The idea of the expert opinion was conceptualized by the first three authors of this paper. These experts then identified the other experts in the field of cardiology, endocrinology, and Lipidology. Each expert was identified to have over 10 years of experience in their respective field. These experts were then invited for digital meetings to discuss the evidence and current role of BA in dyslipidemia (ASCVD) management and the opinions of all the experts were collected in the meetings. A total of five consecutive digital meetings involving 91 experts were held for obtaining expert opinions.

4. Expert opinions

4.1. Lipid Management - Which Guidelines are More Suitable for Indian Patients?

There are multiple national and international guidelines that provide recommendations for the effective management of dyslipidemia. The European [1] and American [2] guidelines are widely adopted across the globe. In India, the Lipid Association of India (LAI) has published its consensus recommendations for the management of dyslipidemia [27]. These guidelines differ from other international guidelines in that they have created an additional group of ASCVD risk, the “extreme-risk” group, besides low, high, and very-high-risk groups. In this extreme-risk group, patients with coronary artery disease (CAD) with three additional CV risk factors are considered. The LDL-C target advised for this group is <30 mg/dl. Thus, with the availability of multiple guidelines, there may be a dilemma among physicians as to which guidelines to adopt to the fullest extent. Some experts indicated that there might be lower acceptance of LAI consensus recommendations at the pan-India level. The reasons for this could be multiple. Firstly, since LAI consensus recommendations were released in the recent past, its adoption on the pan-India level might take more time. Secondly, they have not been supported by national authorities, which could again impact their immediate adoption. Nonetheless, some experts believe the LAI consensus recommendations are more suited for the Indian setting. In our opinion, irrespective of which guidelines a physician adopts in routine practice, the focus should be on optimal treatment with the achievement of LDL-C targets.

Expert opinion: amidst the availability of multiple guidelines, LAI consensus recommendations may be more suitable in the Indian setting.

4.2. Achieving LDL-C Target With High-Intensity Statins (HIS)

The presence of ASCVD assigns the high CV risk status to the individual. Any patient with existing ASCVD is recommended to be treated with HIS. The American College of Cardiology/American Heart Association (ACC/AHA) guidelines on cholesterol management have recommended HIS or maximally tolerated statin therapy to all patients with (i) existing clinical ASCVD, (ii) those with LDL-C levels of 190 mg/dl or more, and (iii) diabetic patients aged 40-75 years with LDL-C levels ≥70 mg/dL [2].

A study from the United States identified that among the ASCVD disease spectrum, patients with CAD were more likely to receive HIS and remained adherent to it compared to patients with stroke or transient ischemic attack (TIA), and peripheral arterial disease (PAD) [28]. Similar trends have been reported in a study from China. Also, the overall prescription rate of statins in ASCVD patients at discharge was below 60% [29]. In assessing the prescribing of HIS after ASCVD, Romanelli et al. reported that HIS was initiated only in 16.6%. The rate of intensification of statin therapy over the mean follow-up period of 16.8 months was 8.4 per 100 person-years [30]. The DA VINCI study from the European Union observed that among patients in the very high-risk primary and secondary prevention groups, HIS monotherapy was used in 20% and 38%, respectively [31]. In India, a study by Das et al. identified that among patients of type 2 diabetes (n=4002) with ASCVD and ASCVD risk factors, 87.2% and 77.9% were not receiving statin at the recommended doses [32]. These data indicate that HIS is not optimally prescribed in ASCVD.

Expert opinion: the majority of patients with clinical ASCVD may remain on a sub-optimal dose of statin. Statin intensification among patients with clinical ASCVD in the Indian setting still remains a challenge.

4.3. Achieving LDL-C Target With Add-ons to Statin Therapy

**Figure 1:**
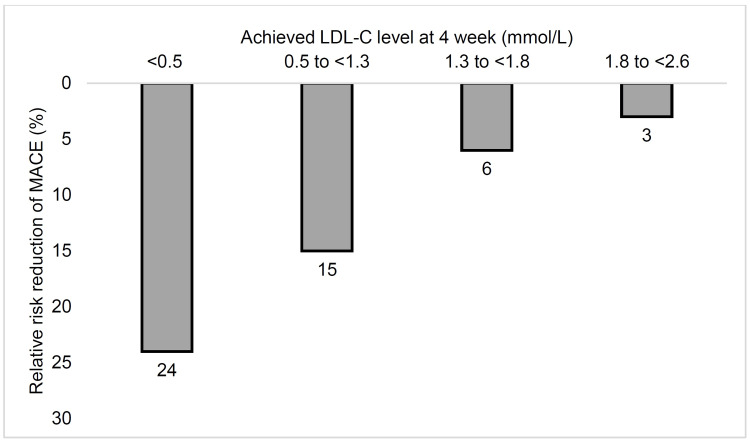
Relative risk reduction with achieved LDL-C levels in the FOURIER trial LDL-C: low-density lipoprotein cholesterol

Achieving the LDL-C target is important for all patients with ASCVD or HeFH or those at high risk of MACE. A recent analysis of the FOURIER trial over a median period of 2.2 years demonstrated incremental benefits in terms of relative risk reduction (RRR) of MACE with reduction of LDL-C achieved at four weeks with evolocumab used as an add-on to statin therapy (Figure 1). The achieved lower LDL-C level was not associated with any safety concerns [33]. An analysis of the IMPROVE-IT trial reported that the primary composite endpoint [comprising CV death, myocardial infarction (MI), unstable angina (UA) requiring hospitalization, coronary revascularization >30 days after randomization, or stroke] was significantly lower with achieved LDL-C levels at one month with simvastatin/ezetimibe combination than simvastatin alone. Compared to achieved LDL-C of ≥70 mg/dl [hazard ratio (HR): 1.00), LDL-C achievement of 50-69 mg/dl, 30-49 mg/dl, and <30 mg/dl were associated with HRs of 0.82, 0.80, and 0.79, respectively. In terms of safety, the study did not find safety concerns in any of the nine prespecified events including myopathy/myalgia/creatine kinase elevation, rhabdomyolysis, neurocognitive events, and others [34].

Despite clear evidence of achieving benefits with LDL-C lowering, the majority of patients may not achieve the LDL-C targets. In a cross-sectional study among patients with diabetes from four centers in India, Mithal et al. [35] reported the achievement of the LDL-C target of <100 mg/dl in 48.74% of patients. In contrast, among patients with coexisting overt CV disease, an LDL-C target of <70 mg/dL was achieved only in 22.87% of patients. In the DA VINCI study, LDL-C goal attainment rates as per 2019 ESC/EAS guidelines among very-high-risk primary and secondary prevention patients were 17% and 22%, respectively. The combined use of ezetimibe or any LLT with PCSK9 inhibitor was associated with 2019 goal attainment rates of 20% and 58%, respectively [30]. In a study of a cohort of CAD patients from Sweden, Mazhar et al. [36] observed that despite an increase in the proportion of patients receiving HIS from 12% in 2012 to 78% in 2018 and remaining adherent (over 80% adherence), the LDL-C goal attainment rates remained at 52% at one year and <50% in subsequent years. These findings are further reinforced by observations from the International Cholesterol Management Practice Study (ICLPS) that was conducted in 18 countries in Eastern Europe, Asia, Africa, the Middle East, and Latin America. At enrolment, only 25.3% were on HIS. Among the very-high-risk, high-risk, and moderate-risk patients, only 32.1%, 51.9%, and 55.7% achieved their LDL-C goals, respectively [37].

Expert opinion: despite administering HIS or statin with other LLTs, a notable proportion of patients with ASCVD may not achieve the LDL-C targets. The proportion of such patients varies vastly across different populations. In Indians, nearly 30% of ASCVD patients may not achieve LDL-C targets, indicating the requirement for the optimization of LLT.

4.4. Statin Intolerance

SI is the inability to tolerate statins due to the development of side effects (such as myopathy), or changes in blood parameters (such as liver enzymes) causing concern with the intake of statins. It can be partial (occurring at higher doses) or complete (at all doses). SAMS occur in nearly 15% of cases whereas rhabdomyolysis is rarer, occurring in one in 12 million people. Factors such as older age, female gender, Asian ethnicity, coexisting neuromuscular conditions, liver or kidney disease, family history of myopathy, excessive exercise, excess alcohol intake, HIS, and drug interactions (such as gemfibrozil, macrolide antibiotics, azole antifungals, etc.) are associated with SI [38]. A meta-analysis of 176 studies with 112 RCTs and 64 cohort studies reported the prevalence of SI to be 9.1% and it was comparatively lower in RCTs than in cohort studies (4.9% vs. 17%, respectively) [26]. There is a relative dearth of studies assessing SI in the Indian context. A cross-sectional survey of 404 clinicians from 23 cities in India reported that 92% found SI in nearly 20% of their patients in routine clinical practice [24].

Expert opinion: the rate of SI in routine clinical practice is <10% and may be at a maximum level of 20% in some select high-risk groups.

4.5. Non-Statin Options for Achieving LDL-C Targets

Statins are primary LLTs for achieving LDL-C lowering. In ASCVD patients who do not achieve the LDL-C target with HIS, the other add-on LLTs include fibrates, ezetimibe, PCSK9 inhibitors, BA, and bile acid sequestrants. The choice of non-statin agents may depend on various factors. In ASCVD patients, a non-statin drug is advised when additional LDL-C lowering is necessary for a patient on maximally tolerated statin therapy. The recent ACC/AHA 2022 focused update suggests that the choice between these drugs be based on control of other risk factors, clinician-patient decision, costs, and statin-related side effects in addition to the percentage of LDL-C reduction required. The choice of non-statin agents is directed by the CV risk. As for very-high CV risk in an existing ASCVD patient, consider ezetimibe and PCSK9 inhibitors for LDL-C reduction of <25% or >25%, respectively [39]. BA is another non-statin option that has been recently approved. In a pooled analysis [17] of four BA trials, the mean LDL-C change in patients with ASCVD, HeFH, or both and those with SI was significant with BA when compared to placebo (difference between the groups: -17.8% and -24.5%). In a cross-sectional survey of physicians in India, 52.4% preferred adding ezetimibe to maximally tolerated statin therapy for LDL-C target attainment [24]. The ACC/AHA update indicates that BA can be an option in ASCVD patients who are at very high as well as high risk of CV disease [39]. In the Indian context, BA and ezetimibe are both suitable options as add-ons to statins. A recent LAI consensus recommends the initiation of BA after ezetimibe among patients with ASCVD [40].

Expert opinion: oral non-statin therapies are preferred options as an add-on to maximally tolerated statin therapy to further lower LDL-C levels in patients with ASCVD.

4.6. Pleiotropic Effects of Bempedoic Acid

The current evidence with BA clearly indicates that besides LDL-C reduction, it reduces hsCRP levels. hsCRP is an established inflammatory marker that has been shown to be of prognostic value in patients with CAD [41-43]. Elevated hsCRP is an evidence-based, highly reproducible risk marker both in primary and secondary prevention of ASCVD. For targeting hsCRP, clinicians have the unique opportunity to use non-statin drugs like ezetimibe and bempedoic acid on top of statins. With PCSK9i, there is an exception as they do not have any effect on hsCRP levels irrespective of PCSK9 monoclonal antibody types [44]. Thus, BA can be considered for effective hsCRP reduction along with statins. In addition, with BA, there is no increase in new-onset or worsening of diabetes, and a mild reduction in HbA1c is observed. This can be potentially beneficial in clinical settings. Ezetimibe is another oral non-statin therapy that is also useful in patients with diabetes mellitus [45]. Regarding BA, a pooled analysis of four phase-III trials revealed that the HbA1c reduction in the BA group was -0.12% and -0.06% in patients with diabetes and prediabetes, respectively [18]. Also, the reduction in hsCRP and ApoB is significant with the use of BA.

Expert opinion: hsCRP being a marker of CAD, a significant reduction of hsCRP offered by BA is an added advantage, along with no worsening of glycemia, in ASCVD patients.

4.7. Hyperuricemia and Bempedoic Acid

With the use of BA, serum uric acid levels tend to rise as the BA glucuronide metabolite competes with uric acid for the same renal OAT2 transporter. Clinical evidence indicates a significant increase in uric acid levels, especially in patients with gout in whom acute gout was precipitated with BA use. Thus, patients with a history of gout or those with elevated uric acid levels need to be monitored with heightened vigilance [9]. In patients without a history of gout and uric acid levels within normal limits at baseline, the risk of gout in subsequent periods is minimal. Additional monitoring of urate levels may be necessary for select suspected groups of patients.

Expert opinion: the cumulative risk of hyperuricemia and risk of gout precipitation as seen in trials with BA is not very high and should not be a limiting factor while using BA. With the appropriate patient selection, the risk of hyperuricemia can be mitigated.

5. Discussion

BA is a novel oral, non-statin LLA that effectively reduces LDL-C, non-HDL-C, ApoB levels, and hsCRP levels in patients with clinical ASCVD or HeFH. It is a new and useful addition to the lipid management armamentarium that is effective and safe when used as an add-on to statins. India enjoys a unique opportunity to meet the tough targets recommended by guidelines where off-patented medications like BA are made available at economical prices.

**Figure 2:**
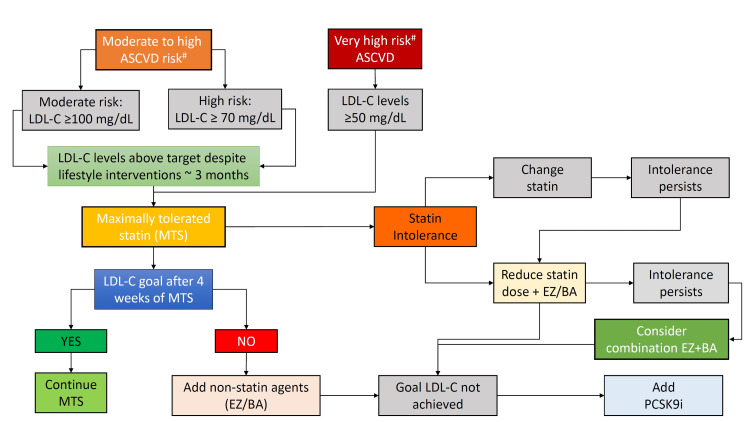
Schematic representation of the current place of bempedoic acid in lipid management MTS: maximally tolerated statin; ASCVD: atherosclerotic cardiovascular risk; EZ: ezetimibe; BA: bempedoic acid; LDL-C: low-density lipoprotein cholesterol; PCSK9i: proprotein convertase subtilisin/kexin type 9 inhibitor

In the Indian context, despite the availability of statins, they are not used in adequate doses in patients with ASCVD. The reasons for this may be manifold but one of the important reasons is the SAMS-associated HIS therapy. In such cases, wherein appreciated SAMS risk is higher, a lower dose of statin may be preferred with the addition of BA to achieve the LDL-C targets. It is essential to diagnose true SI in the clinical setting. In statin-intolerant cases, non-statin therapies, primarily BA and ezetimibe, are essential for lipid management. The combination therapy of BA with ezetimibe is also identified to be cost-effective [46]. The use of PCSK9i may be limited in Indian settings given the concerns of affordability and availability. New-onset diabetes mellitus is a risk with the use of LLAs and it goes hand-in-hand with an increase in the dose of statins. BA is not associated with an increased risk of worsening glycemia or the development of new-onset diabetes. Instead, a slight reduction in glycemic levels is seen with BA (mean percent reduction in HbA1c: -0.12%), which can be beneficial. In diabetic patients, this effect in combination with lipid-lowering and reduction of inflammatory markers as indicated by hsCRP reduction can provide substantial benefits. BA is a relatively safer LLA. The risk of hyperuricemia is minimal in patients with normal baseline uric acid levels. Routine monitoring of serum uric acid levels is necessary only in a select group of patients who are at higher risk of hyperuricemia. It is important to note that diabetes is a major risk factor for ASCVD and is present concomitantly in a majority of patients with ASCVD. Sodium-glucose co-transporter 2 inhibitors (SGLT2i) are one of the first-choice oral antidiabetic drugs (OADs), which reduce serum uric acid levels significantly and are identified as a class effect [47]. Concomitant use of SGLT2i along with BA in appropriate patients might be beneficial in tackling this side effect. Thus, the choice of BA as an LLA can be useful as an add-on to statins for ASCVD or HeFH patients. It is also a beneficial choice for use in combination with ezetimibe in statin-intolerant cases. Through its lipid-lowering activity and its pleiotropic benefits, BA can offer effective cardiovascular protection in the Indian population. Figure 2 schematically provides the current status/place of BA in the armamentarium of lipid management.

## Conclusions

BA, by virtue of its mechanism of action, leads to LDL-C reduction in addition to statin. Given the same pathway of action of statins and BA, it may prove to be more effective as an add-on to statins in terms of LDL-C reduction. BA has shown great promise in LDL-C reduction, hsCRP reduction, and no increase in the occurrence of new diabetes or worsening of glycemia. BA offers substantial clinical benefits except for limited use in known cases of gout. In the Indian context, BA offers another suitable alternative for ezetimibe to be considered as an add-on to statins in ASCVD patients or to be used in combination with other LLTs in statin-intolerant cases.
